# Inverse Association of p63 Expression with Hormone Receptor Status in Invasive Breast Cancer

**DOI:** 10.3390/jpm16070359

**Published:** 2026-07-01

**Authors:** Panagis Lykoudis, Maria Papadoliopoulou, Alexios Kozonis, Georgios Kirkilesis, Marios-Konstantinos Tasoulis, Mahrokh Nohadani, Mihir A. Gudi

**Affiliations:** 1 Department of Pathology, Imperial College London, London SW2 1NY, UK; 2 Attikon University Hospital, National and Kapodistrian University of Athens, Rimini 1, 12462 Chaidari, Greece; 3 Breast Surgery Unit, The Royal Marden NHS Foundation Trust, London SW3 6JJ, UK; 4 Division of Breast Cancer Research, The Institute of Cancer Research, London SW3 6JB, UK; 5 Hammersmith Hospital, Imperial College London, London SW7 2BX, UK; 6 Department of Pathology and Laboratory Medicine, KK Women’s and Children Hospital, 100 Bukit Timah Road, Singapore 229899, Singapore

**Keywords:** p63 gene expression, breast cancer, luminal type

## Abstract

**Background/Objectives**: Immunohistochemistry is an integral component of the diagnostic approach in breast cancer and remains essential for tumor characterization and therapeutic decision-making. p63 gene expression may have potential diagnostic and prognostic roles in breast cancer patients. **Methods**: In this study, 127 specimens of invasive breast carcinoma and 50 control cases were evaluated for p63 gene expression and compared to other pathology factors. **Results**: None of the 50 control cases was assessed as positive for p63 expression. Progesterone and estrogen receptor status were the only factors that demonstrated a statistically significant negative correlation with p63 expression (*p* = 0.005 and *p* = 0.017, respectively). Tumor size demonstrated a marginally non-significant correlation with p63 expression (*p* = 0.051). None of the remaining factors was significantly correlated with p63 expression. **Conclusions**: In conclusion, p63 expression is inversely correlated with estrogen and progesterone receptor status, and type, size and grade of the tumors are not correlated with the gene’s expression, nor is HER2 status. This conclusion might impact genotype-based stratification pertinent to diagnosis and tailored treatment.

## 1. Introduction

Breast cancer is the most common malignancy in women, with approximately 1 in 8 women expected to be affected during their lifetime, and it remains a leading cause of cancer-related mortality worldwide. In the United Kingdom, it is the most frequently diagnosed cancer, with an average of 58,253 new cases and 11,200 deaths annually between 2022 and 2024 [1]. According to the National Cancer Institute of the United States, 316,950 new cases and 42,170 deaths were expected in 2025 [2].

Beyond its financial burden, breast cancer has a profound social and psychological impact, as it affects an organ closely associated with femininity. The loss of the breast, associated grief, and altered body image can significantly affect psychological well-being, while physical consequences such as pain, discomfort, and numbness further impair quality of life. Consequently, a major focus of medical research is to improve disease outcomes through better understanding of risk and prognostic factors, earlier diagnosis, optimized treatment strategies, and improved management of advanced disease.

As in many diseases, research has increasingly focused on genetic and subcellular mechanisms. The identification of Human Epidermal Growth Factor Receptor 2 (HER2) in breast cancer and c-kit in hematologic malignancies marked a turning point in oncology, not only due to their biological significance but also because they became effective therapeutic targets, revolutionizing treatment outcomes [3]. Since then, numerous genes and proteins have been investigated, expanding our understanding of cancer biology. Among these, p63 has recently attracted attention as a potential biomarker in breast cancer.

p63 is located on chromosome 3q27–29 and shares structural similarity with the well-known p53 protein. It encodes six different isoforms based on alternative N-termini (TA and ΔN) and C-termini (α, β, γ) [4], resulting in proteins ranging from 44 to 72 kDa. It is primarily expressed in the nuclei of basal cells of stratified and transitional epithelia. Functionally, knockout studies have demonstrated its essential role in the development of squamous epithelium, as well as mammary and salivary glands [5]. Depending on the isoform, p63 can act either as a tumor suppressor (TA isoforms) or as an oncogene (ΔN isoforms) [6].

Early studies have demonstrated that p63 expression in the breast is largely confined to myoepithelial cells [7]. Subsequent investigations, such as that by Nylander et al. [8], have revealed overlapping expression patterns of the isoforms. In normal breast tissue, ΔNp63 predominates in myoepithelial cells, whereas TAp63 has been reported in malignant cells. However, other studies have suggested that ΔNp63 is the dominant isoform in invasive carcinomas [9,10], highlighting ongoing controversy.

Further functional insights were provided by Senoo et al. [6], who showed that TAp63γ downregulates vascular endothelial growth factor (VEGF) while ΔNp63α upregulates it. These findings suggest a role for p63 in tumor progression and angiogenesis. Importantly, since myoepithelial cells are typically absent in invasive carcinomas, the absence of p63 expression has been proposed as a marker of invasion.

Several studies have evaluated p63 as a diagnostic tool, particularly in fine-needle aspiration biopsies (FNABs). While early studies showed limited utility [9], subsequent research demonstrated improved diagnostic accuracy when p63 was combined with other myoepithelial markers [11,12]. Additionally, p63 expression has been investigated across breast cancer subtypes, with conflicting results. Some studies report higher expression in invasive ductal carcinomas [13,14,15], while others associate it with metaplastic carcinomas [16]. Associations with BRCA1 and HER2 status have also been explored [17,18], along with potential predictive value for response to platinum-based chemotherapy [18,19].

Taken together, these findings suggest that p63 may have both diagnostic and therapeutic relevance in breast cancer [20]. Therefore, investigating its expression across different breast cancer phenotypes may provide further insight into its role as a biomarker.

p63 expression is most commonly assessed using immunohistochemistry (IHC), typically with the 4A4 antibody, which recognizes a central domain common to all isoforms [8,10]. Isoform-specific antibodies, such as KN-TA and KN-ΔN, are available but are primarily used when isoform differentiation is required [8]. It should be noted, however, that IHC results may vary depending on methodological factors and protocol differences [21].

This study aimed to investigate p63 expression in invasive breast carcinoma and to assess its correlation with established prognostic factors, including tumor size, grade, histological subtype, and hormone receptor status.

## 2. Materials and Methods

The archived histopathology reports of a single department were accessed, looking for breast tissue specimens between February 2009 and February 2010. In February 2009, data that were collected were bibliographic and archived histopathological reports for the composition of the Ethics Approval application. Some of these data were maintained for the analysis. Specimen retrieval and histopathological examination (original raw data of this study) and retrieval of other data (clinical outcomes, etc.) were started in June 2009, following notification of Ethics Approval.

### 2.1. Sample Selection

Archived histopathology reports from a single department were reviewed to identify breast tissue specimens over a one-year period. More than 1000 reports were initially retrieved; however, cases involving benign disease, non-invasive carcinomas, and specimens from fine-needle aspiration (FNA) or core biopsies were excluded.

In instances where multiple reports existed for the same tumor (e.g., wide local excision followed by mastectomy), the specimen from the initial excision was selected. In cases of bilateral breast cancer, synchronous or metachronous tumors of non-ductal histology were considered as separate cases.

A total of 135 eligible cases were identified. Receptor status was obtained either from excision specimens or prior core biopsies, as receptor analysis is not routinely repeated unless clinically indicated. All selected cases were reviewed by a single consultant pathologist to confirm the original diagnosis.

Of the 135 identified tissue blocks, 8 were unavailable, resulting in a final cohort of 127 invasive breast carcinoma cases. Additionally, 50 control samples were included from non-invasive areas of the same cohort to validate methodology.

In total, 177 paraffin-embedded tissue blocks were retrieved following approval from the Trust’s Tissue Management Committee and ethical approval from the Royal Ethical Committee of Wales.

### 2.2. Immunohistochemistry Staining

The only primary data collected in this study was p63 expression. In addition to existing clinical data, p63 expression was assessed specifically for the purposes of this study. Given the absence of a universally accepted immunohistochemical scoring system and the expectation of weak expression in invasive carcinomas, the p63 staining was classified as negative (no nuclear staining in invasive tumor cells) or positive (any degree of nuclear staining).

Secondary data included tumor size, patient age, tumor grade and histological subtype, estrogen receptor (ER), progesterone receptor (PgR), HER2 status, and prior chemotherapy exposure. These data were extracted from pathology reports and validated by the same consultant pathologist.

All specimens were received fresh and processed promptly to ensure optimal fixation. Sentinel lymph node specimens, when present, were incised fresh and subsequently fixed in formalin. All tissues were fixed in 4% neutral buffered formalin and embedded in paraffin.

Sections of 3–4 μm thickness were cut using a Shandon Finesse 325 microtome (Thermo Fisher Scientific, Waltham, MA, USA) and mounted on poly-L-lysine–coated slides. The slides were dried overnight at 37 °C.

Paraffin sections were dewaxed in xylene, rehydrated through graded alcohols, and subjected to antigen retrieval in 10 mM citrate buffer using heat treatment. Endogenous peroxidase activity was blocked using 3% hydrogen peroxide in methanol. Non-specific binding was minimized using normal serum.

Immunohistochemical staining was performed using a streptavidin–biotin peroxidase system. The primary antibody used was p63 (clone 4A4, Santa Cruz Biotechnology, Dallas, TX, USA), incubated at the appropriate dilution. Detection was achieved using DAB chromogen followed by hematoxylin counterstaining.

Slides were evaluated under light microscopy (Olympus BX51) at multiple magnifications. Assessment focused exclusively on invasive tumor areas and was performed using a semi-quantitative H-score approach except for p63 evaluation.

### 2.3. Data Analysis

Statistical analysis was performed using SPSS version 20.0 (SPSS Inc., Chicago, IL, USA).

Categorical variables were analyzed using chi-square or Fisher’s exact tests, as appropriate. Variables with multiple categories (e.g., receptor status) were also examined using dichotomized groupings. For twofold tables of categorical variables, the 2-tailed Fisher’s exact test was used. For the rest of the categorical comparisons, the 2-tailed chi-square was used. For scale variables (tumor size and patient’s age), the Mann–Whitney U test was used.

Continuous variables (tumor size and patient age) were analyzed using the Mann–Whitney U test. A *p*-value of <0.05 was considered statistically significant.

## 3. Results

**Figure 1:**
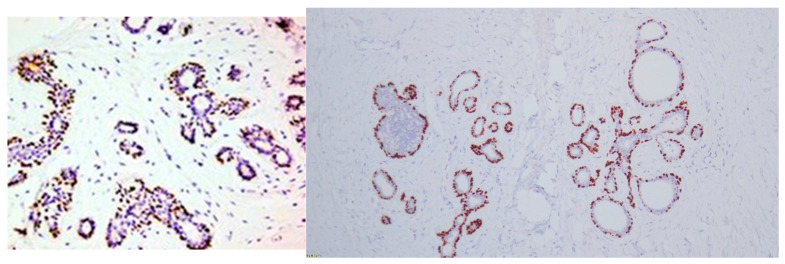
p63 staining highlighting nuclei of myoepithelial cells serving as an internal control. IHC 20× magnification.

**Figure 2:**
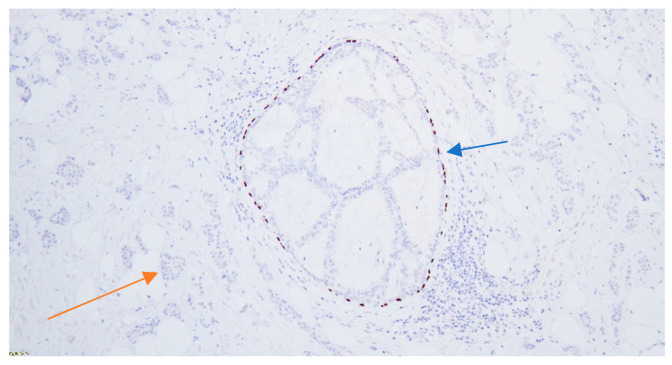
Ductal carcinoma in situ (DCIS) by blue arrow, with negative nuclear staining and only positive rimming by p63-positive myoepithelial cells, and adjoining invasive carcinoma (orange arrow) with no p63 staining. IHC 20× magnification. Both carcinoma components (DCIS and invasive) are considered negative.

**Figure 3:**
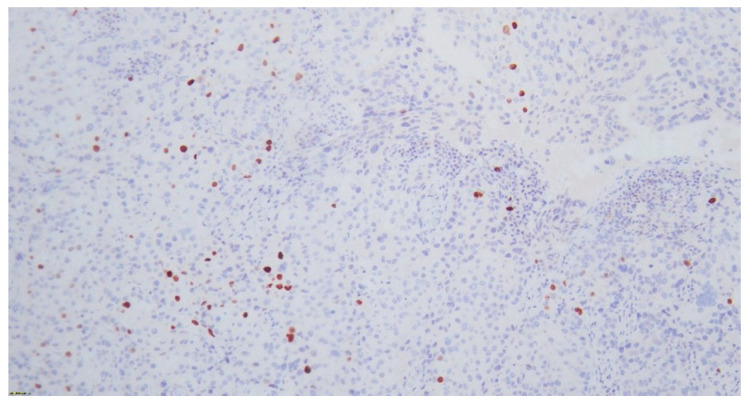
Invasive ductal carcinoma showing positive nuclear staining in the invasive carcinoma cells considered positive. IHC 40× magnification.

None of the 50 control cases was evaluated as positive for p63 gene expression under immunohistochemistry. Out of the 127 invasive breast cancer specimens, only 14 were evaluated as positive (11%). Table 1 presents their characteristics, and in almost all of these cases, nuclear staining was weak, as shown in Figure 1. Table 2 presents the characteristics of the 127 assessed specimens, as well as the *p*-values for each comparison. Age was not statistically significantly correlated with p63 expression. Tumor size was not statistically significantly correlated with p63, but the marginal *p*-value of 0.051 suggests that a statistical significance might have been missed, while median size values across compared groups suggest that tumors expressing p63 might be detected when larger in size. Tumor type was not statistically significantly correlated with p63 expression, neither as a total nor when the ductal and lobular types were compared with the remaining types. The present study included only one case of metaplastic breast cancer and that was positively stained for p63. Tumor grade and previous exposure to chemotherapeutic agents did not seem to affect p63 expression (Table 3 and Table 4, Figure 2 and Figure 3).

Comparison of p63 expression across all four possible groups of estrogen receptor status—namely negative, 1+, 2+ and 3+, according to the H-scoring system—did not yield a statistically significant result. However, when stratifying the cases in ER-negative vs. ER-positive, as well as ER-negative/weakly positive vs. strongly positive, using the H-scoring system, the result was statistically significant, suggesting that the expressions of ER and p63 negatively correlate to each other. Similar were the findings concerning PgR status. Comparison across all four groups was statistically significant itself, and according to further analysis in subgroups, statistically significant negative correlation was found when comparing PgR-strongly positive tumors with the rest of them, as well as PgR-negative and -weakly positive tumors with -moderately and -strongly positive tumors. HER2 expression was not found to be statistically significantly correlated to p63 expression, either as a total or in subgroups. Similarly, triple-negative tumors and ductal triple-negative tumors did not demonstrate more frequent expression of p63 (Figure 1 and Figure 2).

**Table 2:** p63 expression across different histological types of tumors.

		p63 Expression			*p*-Value
		Total, *n* (%)	Negative, *n* (%)	Positive, *n* (%)	
Type	Ductal	103 (81.1%)	92 (81.4%)	11 (78.6%)	0.228
	Lobular	14 (11%)	13 (11.5%)	1 (7.1%)	
	Tubular	3 (2.4%)	2 (1.8%)	1 (7.1%)	
	Papillary	2 (1.6%)	2 (1.8%)	0 (0%)	
	Mucinous	1 (0.8%)	1 (0.9%)	0 (0%)	
	Cribriform	1 (0.8%)	1 (0.9%)	0 (0%)	
	Metaplastic	1 (0.8%)	0 (0%)	1 (7.1%)	
	Non-epithelial	1 (0.8%)	1 (0.9%)	0 (0%)	
	Lymphoepithelioma-like carcinoma	1 (0.8%)	1 (0.9%)	0 (0%)	
Ductal	Non-ductal	24 (18.9%)	21 (18.6%)	3 (21.4%)	0.728
	Ductal	103 (81.1%)	92 (81.4%)	11 (78.6%)	
Lobular	Non-lobular	113 (89%)	100 (88.5%)	13 (92.9%)	1
	Lobular	14 (11%)	13 (11.5%)	1 (7.1%)	
Grade	I	19 (15.1%)	18 (16.1%)	1 (7.1%)	0.513
	II	75 (59.5%)	67 (59.8%)	8 (57.1%)	
	III	32 (25.4%)	27 (24.1%)	5 (35.7%)	
Grade I, II vs. III	I and II	94 (74.6%)	85 (75.9%)	9 (64.3%)	0.344
	III	32 (25.4%)	27 (24.1%)	5 (35.7%)	
Grade I, vs. II, III	I	19 (15.1%)	18 (16.1%)	1 (7.1%)	0.692
	II and III	107 (84.9%)	94 (83.9%)	13 (92.9%)

**Table 3:** p63 expression across groups of different levels of expression of ER, PgR and HER2.

		p63 Expression			*p*-Value
		Total, *n* (%)	Negative, *n* (%)	Positive, *n* (%)	
ER status	−	29 (22.8%)	22 (19.5%)	7 (50%)	0.083
	+	1 (0.8%)	1 (0.9%)	0 (0%)	
	++	11 (8.7%)	10 (8.8%)	1 (7.1%)	
	+++	86 (67.7%)	80 (70.8%)	6 (42.9%)	
ER− vs. any+	−	29 (22.8%)	22 (19.5%)	7 (50%)	0.017
	+, ++, +++	98 (77.2%)	91 (80.5%)	7 (50%)	
ER −/+ vs. ++/+++	−,+	30 (23.6%)	23 (20.4%)	7 (50%)	0.021
	++,+++	97 (76.4%)	90 (79.6%)	7 (50%)	
ER −/+/++ vs. +++	−,+,++	41 (32.3%)	33 (29.2%)	8 (57.1%)	0.065
	+++	86 (67.7%)	80 (70.8%)	6 (42.9%)	
PgR status	−	52 (40.9%)	43 (38.1%)	9 (64.3%)	0.049
	+	13 (10.2%)	11 (9.7%)	2 (14.3%)	
	++	20 (15.7%)	17 (15%)	3 (21.4%)	
	+++	42 (33.1%)	42 (37.2%)	0 (0%)	
PgR− vs. any+	−	52 (40.9%)	43 (38.1%)	9 (64.3%)	0.083
	+, ++, +++	75 (59.1%)	70 (61.9%)	5 (35.7%)	
PgR −/+ vs. ++/+++	−,+	65 (51.2%)	54 (47.8%)	11 (78.6%)	0.045
	++,+++	62 (48.8%)	59 (52.2%)	3 (21.4%)	
PgR −/+/++ vs. +++	−,+,++	85 (66.9%)	71 (62.8%)	14 (100%)	0.005
	+++	42 (33.1%)	42 (37.2%)	0 (0%)	
HER2 status	−	98 (77.8%)	89 (79.5%)	9 (64.3%)	0.327
	+	0 (0%)	0 (0%)	0 (0%)	
	++	8 (6.3%)	6 (5.4%)	2 (14.3%)	
	+++	20 (15.9%)	17 (15.2%)	3 (21.4%)	
HER2– vs. any+	−	98 (77.8%)	89 (79.5%)	9 (64.3%)	0.303
	+, ++, +++	28 (22.2%)	23 (20.5%)	5 (35.7%)	
HER2 −/+/++ vs. +++	−,+,++	106 (84.1%)	95 (84.8%)	11 (78.6%)	0.466
	+++	20 (15.9%)	17 (15.2%)	3 (21.4%)	

ER: estrogen receptor; PgR: progesterone receptor; HER2: Human Epidermal Growth Factor Receptor 2; +++: strongly positive; ++: positive; +: weakly positive; −: negative.

**Table 4:** p63 expression across groups of different combinations of ER, PgR and HER2 expression.

		p63 Expression			*p*-Value
		Total, *n* (%)	Negative, *n* (%)	Positive, *n* (%)	
Triple negative	no	106 (83.5%)	96 (85%)	10 (71.4)	0.247
	yes	21 (16.5%)	17 (15%)	4 (28.6%)	
Ductal triple negative	no	110 (86.6%)	99 (87.6%)	11 (78.6%)	
	yes	17 (13.4%)	14 (12.4%)	3 (21.4%)	0.400

## 4. Discussion

Immunohistochemistry is an integral component of the diagnostic approach in breast cancer and remains essential for tumor characterization and therapeutic decision-making [22,23]. In the present study population, p63 expression was identified in 11% of invasive breast carcinomas without significantly higher expression in a specific subtype. Although high p63 expression has consistently been reported in benign breast lesions, several studies have also associated p63 expression with medullary and metaplastic breast carcinomas [16,24]. However, this was not confirmed in the present study, likely due to the limited representation of such subtypes, as only one case of metaplastic carcinoma was included.

Importantly, this study demonstrated that patient age, tumor grade, and prior exposure to chemotherapy were not significantly associated with p63 expression and therefore are unlikely to act as confounding factors. In contrast, Guo et al. reported an association between younger age, higher histological grade, and p63 expression in HER2-positive breast cancer [22]. These discrepancies may reflect differences in study populations, tumor subtype distribution, or methodological approaches.

One of the most significant findings of the present study is the strong inverse correlation between progesterone receptor (PgR) status and p63 expression, suggesting a biological interaction between these pathways. A similar, though less consistent, inverse association was observed with estrogen receptor (ER) status, particularly when comparing negative or weakly positive tumors with strongly positive ones. These findings support the hypothesis that p63 expression may be more prominent in tumors with basal-like characteristics, which are typically hormone receptor-negative.

No statistically significant association was identified between p63 expression and HER2 status, either in the overall analysis or in subgroup comparisons. These findings are consistent with previously published data [25], although the relationship between p63 and HER2 remains an area of ongoing investigation. The inability of this study to demonstrate correlation between p63 expression and a specific subtype or the HER2 status could be attributed to the relatively small sample size, given the low representation of rarer subtypes in this sample and HER2-positive lesions.

From a diagnostic perspective, numerous studies have explored the role of p63 in distinguishing between benign and malignant lesions, as well as between invasive and non-invasive disease, particularly in fine-needle aspiration biopsy (FNAB) specimens [26,27]. The nuclear localization of p63 provides a technical advantage, as nuclear antigens are generally better-preserved than cytoplasmic markers in such samples. Early studies demonstrated limited diagnostic utility when p63 was used alone [9], but subsequent work showed that its value increases significantly when combined with other myoepithelial markers [28]. Although p63 expression has not proven to be an adequate predictor on its own, combination with other myoepithelial markers seemed to be reliable for this purpose [11,29,30,31].

For example, studies have shown that p63, when used alongside high-molecular-weight cytokeratins or CD10, improves the accuracy of differentiating benign from malignant lesions [12]. Similarly, larger studies have confirmed that p63 is predominantly expressed in benign breast lesions and only rarely in invasive carcinomas, with some exceptions such as papillary subtypes [15]. Additional research has demonstrated variable expression patterns of p63 across different histological subtypes, including adenoid cystic carcinomas [32], invasive ductal carcinomas [17], and metaplastic carcinomas [16].

Beyond its diagnostic utility, p63 has also been implicated in tumor biology and invasion [33,34]. Cheung et al. demonstrated that basal epithelial genes, including p63, are involved in the collective invasion of breast cancer cells [35], while Lewis et al. and Lodillinsky et al. have further supported its role in the transition to invasive disease [36,37]. Moreover, p63 expression has been associated with more aggressive tumor behavior in certain contexts [38,39,40].

Emerging evidence also suggests a potential predictive role of p63 in chemotherapy response. In particular, studies have demonstrated that p63 expression may correlate with increased sensitivity to platinum-based chemotherapy, especially in triple-negative breast cancer [18,19]. However, in the present study, no association was observed between p63 expression and prior chemotherapy exposure, and treatment response was not directly assessed, limiting conclusions regarding its predictive value.

At the molecular level, regulation of ΔNp63 expression has been linked to several pathways and factors, including chromatin-modifying proteins such as SETDB1, TIP60, and histone deacetylases, as well as signaling pathways involving EGFR, Wnt/β-catenin, and STAT3 [41,42,43]. These pathways represent potential therapeutic targets and may help explain the complex role of p63 in tumor biology [44]. Additionally, recent work by Osterburg et al. has proposed a classification of p63 DNA-binding domain mutations, which may further refine its diagnostic and therapeutic relevance in the future [42].

Despite these insights, the present study has several limitations. The relatively small sample size and low prevalence of p63-positive cases limit statistical power and the ability to detect subtle associations. Furthermore, the underrepresentation of certain histological subtypes, particularly metaplastic carcinomas, restricts the generalizability of subtype-specific findings.

Another important limitation is the reliance on immunohistochemistry for assessing p63 expression. While this remains the standard method, it provides only semi-quantitative data and may be influenced by methodological variability [21,45]. More advanced techniques, such as quantitative reverse transcription polymerase chain reaction (qRT-PCR), may offer improved accuracy, although they are not yet fully validated for routine use in this context [46]. However, despite the fact that immunohistochemistry is not considered state-of-the-art, immunostaining remains the basic laboratory method for the study of p63 gene expression [26,37,47]. As shown in the aforementioned studies, the inclusion and use of combination of other myoepithelial markers, such as cytokeratins 5/6, could enhance the studied correlations.

Additionally, the binary classification of p63 expression (positive vs. negative) may oversimplify its biological complexity. Future studies incorporating isoform-specific analysis or quantitative scoring systems may provide deeper insights into the functional significance of p63 expression [47].

Future research should focus on larger multicenter cohorts to validate these findings and better define the role of p63 across different breast cancer subtypes. Particular emphasis should be placed on basal-like and metaplastic carcinomas, where p63 expression may be more relevant. Furthermore, prospective studies evaluating the relationship between p63 expression and response to modern therapies, including targeted and immunotherapeutic agents, would be of significant clinical interest.

In conclusion, p63 expression is relatively uncommon in invasive breast carcinoma but demonstrates a significant inverse association with hormone receptor status, particularly PgR and ER. These findings suggest a potential role for p63 in identifying biologically distinct tumor subgroups. However, its utility as a standalone diagnostic or prognostic marker remains limited, and further studies are required to clarify its role in breast cancer biology and clinical management.

## 5. Conclusions

p63 expression is detected in a subset of invasive breast carcinomas and appears to be inversely associated with hormone receptor status, particularly PgR and ER. These findings suggest a potential role for p63 in identifying specific biological subtypes of breast cancer.

However, its utility as a diagnostic or prognostic biomarker remains limited without further validation. Larger, prospective studies are required to clarify its clinical significance and potential role in guiding therapeutic decisions.

## Figures and Tables

**Table 1:** Characteristics of the 14 p63-positive cases (Size: cumulative size in mm; Chemo: neoadjuvant chemotherapy administered).

Type	Size	Grade	ER	PgR	HER2	Chemo	Age
Ductal	16	II	+++	++	−	No	59
Ductal	50	III	−	−	++	No	85
Ductal	55	II	++	+	+++	No	43
Ductal	10	II	−	−	+++	No	62
Ductal	38	III	+++	−	++	No	59
Ductal	55	III	−	−	+++	No	49
Metaplastic	15	III	−	−	−	No	43
Lobular	60	II	+++	++	−	Yes	62
Ductal	88	II	+++	−	−	No	90
Tubular	19	I	+++	+	−	No	64
Ductal	22	II	+++	++	−	No	72
Ductal	9	II	−	−	−	No	62
Ductal	35	III	−	−	−	No	76
Ductal	33	II	−	−	−	No	67

ER: estrogen receptor; PgR: progesterone receptor; HER2: Human Epidermal Growth Factor Receptor 2; +++: strongly positive; ++: positive; +: weakly positive; −: negative.

## Data Availability

The original contributions presented in this study are included in the article. Further inquiries can be directed to the corresponding authors.
